# A multiparameter approach to monitor disease activity in collagen-induced arthritis

**DOI:** 10.1186/ar3119

**Published:** 2010-08-23

**Authors:** Sylvie Seeuws, Peggy Jacques, Jens Van Praet, Michael Drennan, Julie Coudenys, Tine Decruy, Ellen Deschepper, Lien Lepescheux, Philippe Pujuguet, Line Oste, Nick Vandeghinste, Reginald Brys, Gust Verbruggen, Dirk Elewaut

**Affiliations:** 1 Laboratory for Molecular Immunology and Inflammation, Department of Rheumatology, Ghent University, De Pintelaan 185, Ghent, 9000, Belgium; 2 Biostatistics Unit, Ghent University, De Pintelaan 185, Ghent, 9000, Belgium; 3 Galapagos NV, Generaal De Wittelaan L11 A3, Mechelen, 2800, Belgium

## Abstract

**Introduction:**

Disease severity in collagen-induced arthritis (CIA) is commonly assessed by clinical scoring of paw swelling and histological examination of joints. Although this is an accurate approach, it is also labour-intensive and the application of less invasive and less time-consuming methods is of great interest. However, it is still unclear which of these methods represents the most discriminating measure of disease activity.

**Methods:**

We undertook a comparative analysis in which different measurements of inflammation and tissue damage in CIA were studied on an individual mouse level. We compared the current gold standard methods - clinical scoring and histological examination - with alternative methods based on scoring of X-ray or micro-computed tomography (CT) images and investigated the significance of systemically expressed proteins, involved in CIA pathogenesis, that have potential as biomarkers.

**Results:**

Linear regression analysis revealed a marked association of serum matrix metalloproteinase (MMP)-3 levels with all features of CIA including inflammation, cartilage destruction and bone erosions. This association was improved by combined detection of MMP-3 and anti-collagen IgG2a antibody concentrations. In addition, combined analysis of both X-ray and micro-CT images was found to be predictive for cartilage and bone damage. Most remarkably, validation analysis using an independent data set proved that variations in disease severity, induced by different therapies, could be accurately represented by predicted values based on the proposed parameters.

**Conclusions:**

Our analyses revealed that clinical scoring, combined with serum MMP-3, anti-collagen IgG2a measurement and scoring of X-ray and micro-CT images, yields a comprehensive insight into the different aspects of disease activity in CIA.

## Introduction

The systemic autoimmune disease rheumatoid arthritis (RA) is characterized by synovial inflammation followed by progressive destruction of articular cartilage and subchondral bone. Mouse models are often used to gain further insight into the pathological mechanisms of joint inflammation as well as for preclinical evaluation of therapeutic agents. In this context, collagen induced arthritis (CIA) is the most widely studied animal model for RA as it models the similarities in pathology and immunological processes involved in the disease [1].

Currently, clinical scoring of inflamed paws over time is the standard method used for quantification of disease severity. This is followed by histological examination of joints to assess inflammation, and cartilage and bone loss. More recently, alternative and less time-consuming techniques such as scoring of X-ray and micro-computed tomography (micro-CT) images have been employed. The Larsen score is well established for the scoring of X-ray pictures and accounts for abnormalities of the joint space and bone erosions [2,3]. Alternatively, when performing micro-CT analysis, the *number of objects per micro-CT slice *parameter can be used as an indicator of bone porosity [4].

Moreover, the application of biomarkers that are measurable in serum, urine or tissue has become a topic of increasing interest. Pro-inflammatory cytokines like tumour necrosis factor-α (TNF-α) are detected in arthritic mice and cartilage degradation products such as cartilage oligomeric matrix protein (COMP) and C-telopeptide fragments of type II collagen (CTXII) have been described as potential biomarkers for cartilage destruction in CIA [5-7]. Furthermore, the production of autoantibodies to type II collagen (CII) is a typical feature of CIA [8]. Despite the fact that these methods are regularly used, it remains unclear which read-out parameters are most useful when assessing CIA.

We therefore established an analysis platform in which the degree of inflammation and tissue damage in CIA was assessed at the level of individual mice. This platform incorporated different scoring methods including histological examination and techniques based on scoring of X-ray or micro-CT images. Furthermore, systemically expressed proteins involved in CIA pathogenesis were investigated for their potential as biomarkers.

We report here the findings of our search for useful scoring methods and valuable biomarkers to monitor different aspects of disease severity in CIA. First, we can conclude that a combined analysis of scoring X-ray's using a modified Larsen score and quantification of erosions on the calcaneus as visualized by micro-CT imaging is predictive for both cartilage damage and bone erosions in CIA. Out of the selected proteins in our study, a combined analysis of serum MMP-3 and anti-collagen IgG2a antibody concentrations turned out to be indicative not only for the inflammatory aspect of CIA but also for cartilage and bone destruction, emphasizing the inflammatory nature of the disease. Most important, in an independent experiment, this set of proposed parameters is capable of accurately representing variations in disease severity induced by different therapies. Taken together, this critical analysis of read-outs for monitoring disease activity and therapeutic responses in the CIA model has led to the identification of parameters that allow faster analysis of treatment efficacy with minimal loss of information.

## Materials and methods

### Mice

Male, 9 to 10-week-old DBA/1 mice were purchased from Janvier (Le Genest Saint Isle, France) and housed following institutional guidelines. Experiments were conducted according to the guidelines of the Ethics Committee of Laboratory Animals Welfare of Ghent University.

### Induction and analysis of CIA

Mice were immunized intradermally at the base of the tail with 200 μg of chicken type II collagen (CII) (Morwell Diagnostics GmbH, Zurich, Switzerland) (in 0.1 M acetic acid) emulsified in Incomplete Freund's Adjuvant + mycobacterium Tuberculosis H37RA (150 μg/mouse) (Difco, Lawrence, KS, USA). Twenty-one days later, mice were re-challenged with an injection of CII in Incomplete Freund's Adjuvant. From Day 21, mice were monitored for clinical symptoms of arthritis until the day of sacrifice (Day 42). Clinical severity was graded as follows: 0 = normal; 0.5 = erythema and edema in only one digit; 1 = erythema and mild edema of the footpad, or ankle or two to five digits; 2 = erythema and moderate edema of two joints (footpad, ankle, two to five digits); 3 = erythema and severe edema of the entire paw; 4 = reduced swelling and deformation leading to incapacitated limb.

The individual mouse arthritic score was obtained by summing the scores recorded for each limb. Clinical evaluations were performed by two investigators unaware of mouse identity and the mean of both scores was calculated.

### Semi-therapeutic treatment of CIA

From Day 20 after primary immunization, mice were treated with one of four different therapies: 1) dexamethasone (Organon Laboratories Ltd, Hertfordshire, UK) (1 mg/kg, daily, intraperitoneal injection); 2) etanercept (Wyeth Pharmaceuticals, Louvain-la-Neuve, Belgium) (15 mg/kg, every other day, intraperitoneal injection); 3) zoledronic acid (Novartis, Basel, Switzerland) (100 μg/kg, one single injection on Day 20, subcutaneous injection); 4) abatacept (Bristol-Myers-Squibb, NY, NY, USA) (5 mg/kg, every other day, intraperitoneal). As a control, mice were similarly treated with phosphate buffered saline (PBS).

### Scoring of X-ray images

Prior to histology, anteroposterior X-ray radiographs were taken (Faxitron M20, Edimex, France) of one hind paw per mouse. The severity of bone erosion was blindly ranked by three independent scorers using a modified version of the Larsen scoring method: 0 = normal; 1 = slight abnormality with any one or two of the exterior metatarsal bones showing slight bone erosion; 2 = definite early abnormality with any of the metatarsal or tarsal bones showing bone erosion; 3 = medium destructive abnormality with the metatarsal bones or any one or two of the tarsal bones showing definite bone erosions; 4 = severe destructive abnormality with all the metatarsal bones showing definite bone erosion and at least one of the tarsometatarsal joints being completely eroded, leaving some bony joint outlines partly preserved; 5 = mutilating abnormality with no bony outlines that can be deciphered.

### X-ray microcomputed tomography analysis (micro-CT)

3 D micro-tomodensitometry of calcaneus (Skyscan 11.70, Kontich, Belgium; X-ray voltage: 60 kV; current: 100 μA; 1,000 × 600 large camera resolution) was used to quantify bone erosion within the paws. Image analysis (CTAn software, Skyscan) allowed quantification of the *mean number of objects *per slice.

### Histological evaluation

To allow comparison between the different techniques, histology was performed on the same paws that were used for X-ray and micro-CT imaging. Knees and paws were fixed in 4% formaldehyde, decalcified and embedded in paraffin. Serial sections of the knee were stained with hematoxylin and eosin (H&E) or with saffranin O-fast green and inflammation and joint damage of the femorotibial joint were investigated by scoring five parameters as follows: inflammation was scored on a scale of 0 (no inflammation) to 3 (severe inflamed joint) depending on the number of inflammatory cells in the synovial cavity (exudate) and synovial tissue (infiltrate). Exudate and inflammatory infiltrate were both assigned individual scores. Loss of proteoglycans was scored on a scale of 0 to 3, ranging from fully stained cartilage to destained cartilage or complete loss of articular cartilage. Cartilage destruction was scored on a scale of 0 to 3, ranging from the appearance of dead chondrocytes (empty lacunae) to complete loss of the articular cartilage. Loss of bone was scored on a scale of 0 to 5 ranging from no damage to complete loss of the bone structure [9]. We also stained serial sections of the paw and examined the following parameters: pannus severity, infiltrate, cartilage lesion and bone lesion. The scoring system ranged from 1 to 4: 1 = normal, 2 = mild, 3 = moderate, 4 = severe.

For both knee and paw histology, a composite score was calculated by summing the individual parameters. Scoring was executed blindly by two investigators and mean values were calculated.

### Serum analyses

Serum was collected at the end of the experiments. Standard ELISA kits were used for determination of serum levels of anti-CII specific IgG1 (Chondrex, Redmond, WA, USA) and IgG2a (MD Biosciences, Zurich, Switzerland) antibodies, COMP (AnaMar Medical, Göteborg, Sweden), MMP-3 (R&D Systems, Abingdon, UK) and C-terminal cross-linking telopeptide of type I collagen (CTX I) (RatLaps EIA) and CTX II (Serum Pre-Clinical Cartilaps ELISA) (both from Immunodiagnostic Systems, Boldon Business Park, UK).

Levels of interleukin (IL)-6, IL-17, Keratinocyte chemoattractant (KC) and TNF-α were determined by a Luminex Beadlyte kit (4-plex) (Upstate, VA, USA).

### Statistical analysis

For analysis of longitudinal clinical scores, mixed model analysis with random intercept was used. Differences in clinical (day of onset and Day 42 score) and histological data between the treatment groups were assessed by Kruskal Wallis tests followed by Mann-Whitney-U test with correction using the Holm procedure. Fisher's Exact test was applied to analyse arthritis frequencies. Stepwise linear regression analysis was performed with the scoring methods, serum markers and treatment group as covariates. All analyses were performed using SPSS 15.0 statistical software (Chicago, IL, USA).

## Results

### Evaluation of CIA by clinical and histological examination

**Figure 1:**
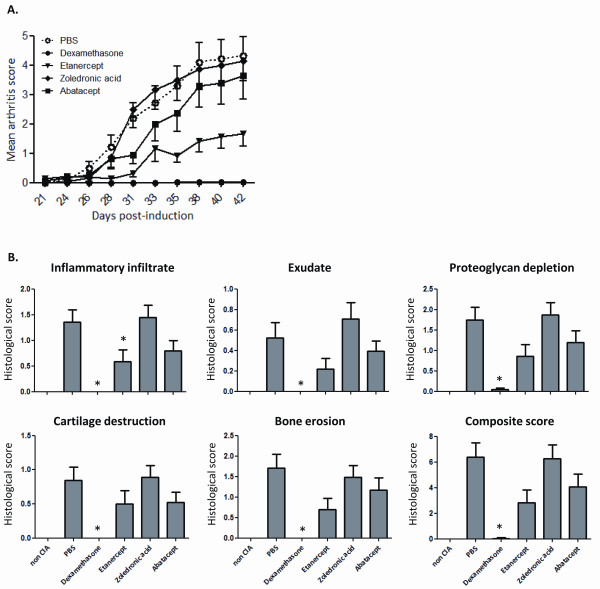
**A) Clinical scoring of arthritis symptoms in CIA**. From Day 20 after CIA induction, mice received semi-therapeutic treatment with one of four therapies: dexamethasone (*n *= 23), etanercept (*n *= 23), zoledronic acid (*n *= 24) or abatacept (*n *= 23). Control mice were treated with the vehicle PBS (*n *= 24). Signs of arthritis were monitored until Day 42. Significant reduced arthritis scores were observed for dexamethasone and etanercept treatment as compared to PBS administration (mixed model analysis, random intercept, *P *< 0.001 for both dexamethasone and etanercept). Means + SEM of pooled data from two independent experiments are depicted. **B) Histological examination of arthritis symptoms in the knee joint of CIA mice**. At Day 42 post-immunization, knee joints were isolated and prepared for histological examination. Femorotibial joints were scored for five different parameters. Kruskall-Wallis test: *P *≤ 0,001 for all parameters as compared to PBS. *: significant *P*-values (compared to PBS) according to Mann-Whitney U test and correction using the Holm procedure. Bars represent means + SEM.

We initiated this study by examining CIA severity using the current gold standard method: clinical scoring followed by histological analysis. To create gradations in disease severity, mice were treated semi-therapeutically with one of four different therapies: dexamethasone, etanercept, zoledronic acid or abatacept. Vehicle (PBS) administration was used as a negative control. Pooled data of two independent experiments are shown in Figure 1A and Table 1. As expected, semi-therapeutic treatment of mice with dexamethasone completely abolished the induction of CIA. Also etanercept induced significant improvement in arthritis symptoms. Treatment with abatacept on the other hand resulted in a small, albeit not significant, reduction in disease severity and one-time administration of zoledronic acid showed no protective effect.

**Table 1:** Overview of pooled clinical data from two individual experiments

		Incidence of arthritis		Day of onset of arthritis			Day 42 score		
				
Treatment groups	N	Frequency of arthritis (%)	*P-*value (Fisher's exact test)	Median	IQR	*P- *value (MW) (compared to PBS)	Median	IQR	*P- *value (MW) (compared to PBS)
Vehicle (PBS)	24	87.5		29	26 to 33		3.25	3 to 6	
Dexamethasone	23	4.3	0.000*	ND	ND	ND	0	0 to 0	0.000*
Etanercept	23	69.6	0.168*	33	23 to 35	0.600	0.5	0 to 3.5	0.004*
Zoledronic acid	24	83.3	1.000*	31	28 to 32	0.212	4.5	0.625 to 6	0.840*
Abatacept	23	73.9	0.286*	28	25 to 32	0.400	3	0 to 7	0.378*

Depicted are the number of mice (N), frequency of arthritis at Day 42, median day of onset and median arthritis score at Day 42 with their interquartile range (IQR) and *P*-values (*: significant *P*-values according to Mann-Whitney U test (MW) and correction using the Holm procedure). Fisher's exact test for frequencies of arthritis: *P *< 0.001; Kruskall Wallis test for day of onset: *P *= 0.349; Kruskall Wallis test for Day 42 score: *P *< 0.001. ND, not determined. PBS, phosphate buffered saline.

In contrast to clinical scoring, which is mainly indicative of the degree of inflammation, histological examination provides additional information pertaining to cartilage and bone damage. In line with the absence of clinical symptoms, histological scoring of the knee joint revealed no signs of inflammation (inflammatory infiltrate and exudate), proteoglycan depletion, destruction of articular cartilage or bone erosion in dexamethasone treated animals (Figure 1B). Although the composite score of knee histology was borderline not significant after statistical correction using the Holm procedure (*P *= 0.019 > 0.017 (Holm)), the attenuated clinical disease activity observed in mice treated with etanercept was in agreement with strong reductions in the five parameters analysed when compared to vehicle treated mice. Comparable to a rather small improvement of clinical arthritic symptoms in the abatacept treated mice, all histological parameters investigated showed a moderate reduction in severity. Treatment with zoledronic acid did not induce improvement in any of the histological factors. In the context of the four therapies used, similar trends were observed between the histological assessment of disease and the clinical score.

Beside the knee joint, also hind paw joints are commonly subjected to histological examination and in the present study, we examined the correlation between both techniques and the clinical score. Based on our results, it can be concluded that although clinical scoring addresses paw inflammation, histological examination of the knee joint correlates slightly better with the clinical scoring as compared to paw histological scoring (r = 0.725 and r = 0.533 for histology composite scores of respectively knee and paw versus clinical scoring on Day 42). These findings highlight the fact that CIA is a systemic disease and that both sites are useful for histological examination.

### Scoring of plain X-ray and micro-CT images are valuable methods to assess cartilage and bone damage in CIA

**Figure 2:**
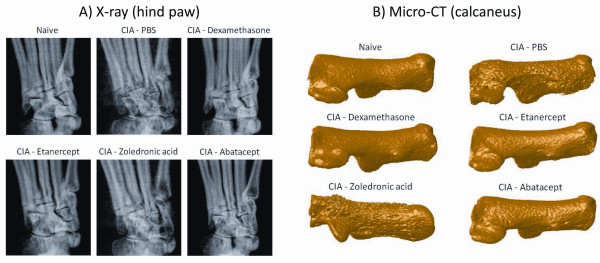
**Visualisation of joint damage by X-ray and micro-CT imaging**. **A)** X-ray pictures of representative paws from naive and treated or untreated CIA mice. **B)** Three-dimensional reconstructions of micro-CT images of representative calcaneus bones from naive and treated or untreated CIA mice.

Despite the fact that histology provides additional information on cartilage and bone damage, the procedure is time-consuming and only allows cross-sectional analysis. We therefore compared the use of two non-invasive scoring techniques based on X-ray and micro-CT imaging of paws, with both clinical and histological scoring. For scoring of plain X-ray images, a modified Larsen score was applied on hind paws of the mice. On reconstructed three-dimensional micro-CT images, the focal bone erosion at the calcaneus is clearly visible (Figure 2). This bone erosion was quantified by counting the average *number of objects per slice *on the 2 D micro-CT images of the calcaneus, using image analysis software.

For further analysis, stepwise linear regression modelling was performed on data from a first experiment. Linear regression analysis, using a clinical score on Day 42 as the dependent variable and composite scores for knee and paw histology, Larsen score, micro-CT calcaneus score and treatment group as independent variables, indicates that from these four different scoring methods, clinical scoring is most accurately represented by the composite score of knee histology (adjusted R^2 ^= 0.464). Despite this strong correlation between the clinical score and knee histology, we opted to investigate the capacity of X-ray or micro-CT imaging, performed on paws, to predict joint damage, in relation to histology of the paw. Therefore, two linear regression analyses with either the cartilage or bone parameter of the paw histology as dependent variable were fitted (Table 2). Results suggested that the Larsen score was most indicative for cartilage destruction in the paw, but the predictive value of the regression model was greatly enhanced by addition of the calcaneus erosion score and the clinical score (R^2 ^= 0.704). However, for the detection of bone erosions, the calcaneus score showed the best correlation with histology yet, combined analysis of calcaneus and Larsen score was much more predictive for bone erosions (R^2 ^= 0.713). These results indicate that a combined analysis of clinical score, X-ray Larsen score and micro-CT calcaneus score provide useful information regarding cartilage and bone destruction in CIA.

**Table 2:** Best-fit models for clinical score, and cartilage and bone destruction predicted by different scoring methods

Dependent variable	Best-fit model	Adjusted R^2^	Significance
Clinical score	Composite score of knee histology	0.464	< 0.001

Cartilage destruction	Larsen score	0.525	< 0.001
	Larsen score + micro-CT calcaneus score	0.667	< 0.001
	Larsen score + micro-CT calcaneus score + clinical score	0.704	< 0.001

Bone destruction	Micro-CT calcaneus score	0.605	< 0.001
	Micro-CT calcaneus score + Larsen score	0.713	< 0.001

Best-fit models for clinical score, and cartilage and bone destruction in the hind paw as proposed by linear regression analysis with scoring methods and treatment group as independent variables. R^2 ^indicates the squared correlation between the observed values of the dependent variable and the predicted values based on the regression model. Adjusted R^2 ^is a modification of R^2 ^that adjusts for the number of covariates in the regression model.

### MMP-3 is related to inflammation as well as cartilage and bone damage in CIA

For the identification of biomarkers to assess disease severity in CIA, the concentrations of 10 serum proteins were assessed at the time of sacrifice. The selected proteins constitute inflammatory mediators and chemokines involved in arthritis, including TNF-α, IL-17, IL-6, MMP-3 and KC. Additionally, indicators of cartilage degradation (COMP and CTXII) and bone damage (CTXI) were determined as well as the presence of antibodies to type II collagen (IgG1 and IgG2a). As serum levels of IL-6 were mostly undetectable, this cytokine was eliminated from further analysis.

Although analysis on the individual biomarker level revealed significant correlations with clinical score, cartilage destruction and bone erosion for five out of nine proteins (COMP, CTXII, CTXI, anti-collagen IgG2a and MMP-3) (data not shown), only three of them contributed significantly in the performed linear regression models. No significant correlations were observed for the investigated cytokines (IL-17 and TNF-α). In three models with clinical score, or cartilage or bone destruction of the knee histology as dependent variables, we incorporated all nine proteins, including the cytokines, and the treatment group (Table 3). Remarkably, measurement of MMP-3 concentrations in serum was found to be most indicative for clinical score. Analysis of histological parameters revealed that MMP-3 is not only a good indicator for the inflammatory aspect of CIA but also for cartilage and bone damage. Addition of anti-collagen IgG2a to the models improved the association with clinical score and bone destruction. Combined detection of both MMP-3 and CTXII on the other hand was more indicative for cartilage destruction.

**Table 3:** Best-fit models for clinical score, and cartilage and bone destruction predicted by different serum markers

Dependent variable	Best-fit model	Adjusted R^2^	Significance
Clinical score	MMP-3	0.414	< 0.001
	MMP-3 + anti-collagen IgG2a	0.499	< 0.001

Cartilage destruction	MMP-3	0.451	< 0.001
	MMP-3 + CTXII	0.485	< 0.001

Bone destruction	MMP-3	0.372	< 0.001
	MMP-3 + anti-collagen IgG2a	0.411	< 0.001

Best-fit models for clinical score, and cartilage and bone destruction in the knee joint as proposed by linear regression analysis with nine serum markers and treatment group as independent variables. MMP-3, matrix metalloproteinase-3.

### Validation of the proposed regression models

**Figure 3:**
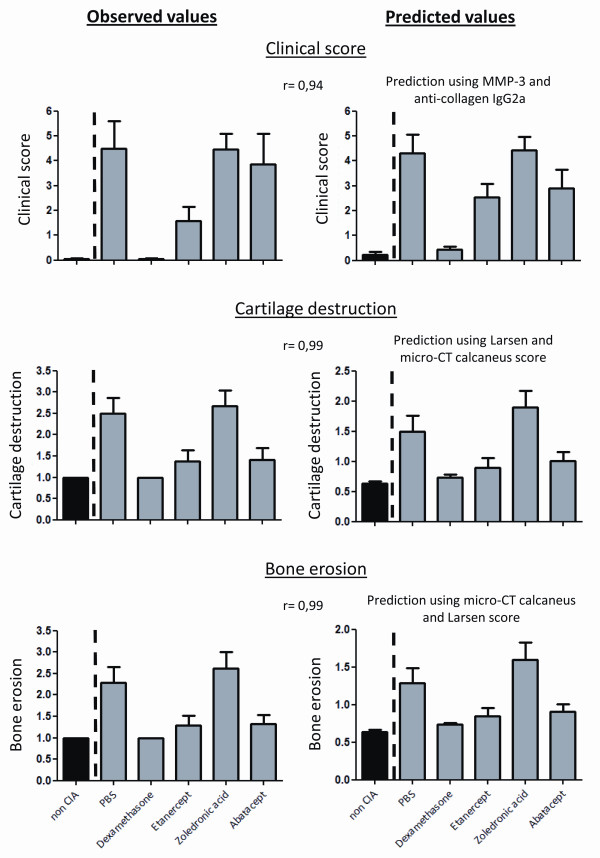
**Validation of the proposed regression models**. Graphical reproduction of the observed and predicted values for clinical score, cartilage destruction and bone erosions based on a second independent data set. Predicted values were calculated using the linear regression equations obtained from the first experiment. Bars represent means + SEM and r = Spearman's rho representing the correlations between the observed and predicted mean values for each treatment group.

To validate the obtained results, the data set from a second independent experiment was fit in the regression equations derived from the initial analyses, resulting in *predicted values*. Because linear regression analysis, combining scoring methods and serum markers, did not yield important contributions for serum markers in predicting cartilage and bone degradation (Table 4), these aspects of the disease were only validated using X-ray and micro-CT analysis. Clinical score on the other hand, was predicted using MMP-3 and anti-collagen IgG2a antibody concentrations. In the validation analyses, we studied the capacity of the predicted values to distinguish between the different therapies. As CIA experiments are always performed on a group of animals and not on the individual level, we opted to perform the validation analysis on the level of the treatment groups. Figure 3 clearly demonstrates that the same trends between the different treatment groups can be detected in the graphical representations of both the observed and predicted values for the clinical score, cartilage degradation and bone destruction. This was confirmed by very strong correlations between the observed and predicted mean values for each treatment group (Spearman's rho = 0.94, R = 0.99 and R = 0.99 for predicted versus observed means of respectively clinical score, cartilage destruction and bone erosions). These validation analyses provides compelling evidence that the proposed read-out parameters, MMP-3, anti-collagen IgG2a, and Larsen and micro-CT calcaneus score, are most applicable to detect changes in different aspects of arthritis severity upon treatment.

**Table 4:** Best-fit models for cartilage and bone destruction predicted by both scoring methods and serum markers

Dependent variable	Best-fit model	Adjusted R^2^	Significance
Cartilage destruction	Larsen score	0.530	< 0.001
	Larsen score + micro-CT calcaneus score	0.664	< 0.001
	Larsen score + micro-CT calcaneus score + clinical score	0.700	< 0.001

Bone destruction	Micro-CT calcaneus score	0.602	< 0.001
	Micro-CT calcaneus score + Larsen score	0.711	< 0.001
	Micro-CT calcaneus score + Larsen score + COMP	0.731	< 0.001

Best-fit models for cartilage and bone destruction in the paw as proposed by linear regression analysis with all scoring methods and serum markers and treatment group as independent variables. COMP, cartilage oligomeric matrix protein.

## Discussion

As in many other human or experimental diseases, the field of surrogate analysis markers is also in RA and its experimental models of increasing importance. Therefore, it is surprising that a comprehensive analysis, aimed at assessing various read-out parameters for monitoring disease activity is still lacking for CIA, the most commonly used animal model of RA. In this report, we use linear regression analysis to propose a restricted set of key indicators that allows the assessment of CIA in a more objective and less labour-intensive way.

When evaluating the value of serum markers, MMP-3 was found to be highly indicative of all the features of CIA, including inflammation, cartilage destruction and bone erosion. This is in line with previous reports that demonstrated elevated MMP-3 expression levels in synovial fluids and sera of patients with rheumatoid arthritis as well as expression in joints of CIA rats [10-12]. Although several studies have proposed MMP-3 as a useful indicator for inflammation in RA, the correlation of MMP-3 with joint destruction or its predictive value is still a matter of debate [10,13-18]. Our results, however, showed a strong correlation of this marker with joint damage. A second marker that also proved to be related to inflammation and bone destruction is the anti-CII IgG2a antibody. Antibodies to CII are detected in both human as well as experimental arthritis early in disease development and the IgG2 subclass is capable of initiating inflammatory processes by activating the complement cascade [8,19-21].

Rather unexpected was the finding that, in combination with MMP-3 and anti-collagen IgG2a, detection of cartilage or bone degradation products such as COMP and CTXI in serum could not improve the predictive value of the regression models for cartilage or bone destruction as assessed by knee histology. However, CIA is an experimentally induced arthritis that is strongly mediated by inflammation. This could clarify why inflammatory mediators rather than specific indicators of cartilage and bone degradation proved to be better biomarkers in this animal model. In addition, since the serum levels for the different markers were assessed at the end of the study, different kinetics of the COMP or CTXI markers might represent another explanation for their lower predictive value observed in this study. This possibility of different kinetics could also provide an explanation for the fact that the inflammatory cytokines TNF-α and IL-17 did not correlate with clinical score and joint damage.

We also evaluated the potential of different imaging techniques to assess disease activity in CIA, which yielded a second set of important findings. First, scoring of X-ray pictures using a modified Larsen score was most indicative for destruction of cartilage. It should be mentioned though that cartilage itself cannot be visualised by X-rays. Therefore, cartilage destruction is quantified indirectly by incorporating joint space narrowing as a parameter in the scoring system. Second, for the assessment of bone erosions, we introduced a rather new method that is based on micro-CT imaging and allows objective and high throughput analyses. So far, bone loss on micro-CT was mainly studied using parameters describing bone volume and composition [22,23]. A major hallmark of RA and CIA, however, is the appearance of focal bone erosions [24] and to quantify these erosions we calculated the *number of objects *on slices through the calcaneus as a measure for bone fragmentation. Our analysis showed that this method correlated well with the bone erosion score of histology. Importantly, in both cases, combined analysis of X-ray Larsen and micro-CT calcaneus score greatly enhanced the predictive values of the regression models for cartilage and bone destruction. Both X-ray and micro-CT imaging allow longitudinal follow-up of disease activity in animals. However, our analyses only take into account the read out-parameters on Day 42 and as such, they do not allow us to draw conclusions about the utility and sensitivity of these methods at earlier time points. A follow-up study comparing the techniques at different time-points of disease progression could answer this question. Additionally, it would also yield useful information about the predictive value of the techniques and it could indicate if a correct analysis for the different aspects of the disease severity in CIA could be obtained at an earlier time-point to shorten the experiment time.

Remarkably, when linear regression analyses, containing both serum markers and scoring methods, were performed, results demonstrated that serum markers did not increase the predictive value of the models for cartilage and bone damage. This implies that serum markers are inferior to imaging methods for the evaluation of disease activity and therapeutic efficacy. One can however not conclude that a role for the investigated serum markers in CIA is minimal. As already mentioned, serum levels of proteins may display certain kinetic patterns in pathologies and as such, timing of the evaluation could be important. Furthermore, serum concentrations of proteins do not necessarily reflect the local situation in the joint. Investigation of biomarker expression in the joint would yield useful information but these methods are often more invasive and as such less applicable.

Validation of the obtained results by utilizing an independent data set, demonstrated that values, predicted by the proposed read-out parameters, were also capable to differentiate between various therapies with a different mode of action. The same trends between the different treatment modalities could be observed in the graphical representations of both the predicted values by Larsen and calcaneus score and the values observed by histological examination. Since the last method is very time-consuming and only allows cross-sectional analysis, combined analysis of X-ray and micro-CT images might provide a worthy alternative. One should, however, also take in mind that the analyses performed in our study focus on the utility of different read-out parameters to assess disease severity in CIA. As different animal models exist to investigate rheumatoid arthritis, for example, adjuvant-induced arthritis, zymosan induced arthritis and so on, a comparable study should be performed for all the other models. Similarly, it would be interesting to investigate the value of imaging techniques and serum markers in other joint disorders, such as osteoarthritis. In other words it remains to be determined whether imaging techniques and serum markers also apply in other models of experimental arthritis.

## Conclusions

We can conclude that MMP-3 is a strong indicator for the inflammatory features of CIA. The fact that this marker is also related to cartilage and bone destruction emphasizes the inflammatory nature of the disease. Furthermore, combined analysis of X-ray Larsen and micro-CT calcaneus erosion score proved to be predictive for both cartilage and bone destruction. Overall, our analyses revealed that clinical scoring, combined with serum MMP-3 and anti-collagen IgG2a measurement and scoring of X-ray and micro-CT images yield a comprehensive insight into the different aspects of disease activity in CIA.

## Abbreviations

CII: type II collagen; CIA: collagen induced arthritis; COMP: cartilage oligomeric matrix protein; CTX I: C-terminal cross-linking telopeptide of type I collagen; CTXII: C-telopeptide fragments of type II collagen; H&E: hematoxylin and eosin; IL: interleukin; IQR: interquartile range; KW: Kruskall Wallis test; Micro-CT: micro-computed tomography; MMP-3: matrix metalloproteinase-3; MW: Mann-Whitney U test; ND: not determined; PBS: phosphate buffered saline; RA: rheumatoid arthritis; SEM: standard error of mean; TNF-α: tumor necrosis factor-α.

## Competing interests

The authors declare that they have no competing interests.

## Authors' contributions

SS, PJ, JVP, MD, LO, NV, RB, GV and DE participated in the study design. SS, PJ, JVP, JC, TD, LP, PP and LO participated in the acquisition of data. ED delivered support for statistical analysis. SS, PJ, JVP and DE participated in the analysis and interpretation of the data. SS and DE prepared the manuscript. All authors read and approved the final manuscript.
